# Case of Inguinal Hernia, in Which the Operation Was Performed without Opening the Sac

**Published:** 1826-07

**Authors:** 

**Affiliations:** St. Thomas's Hospital.


### CASES OF STRANGULATED HERNIA, *requiring Operation.*

*Case of Inguinal Hernia, in which the Operation was performed without opening the Sac.* Treated by Mr. TRAVERS, at ST. THOMAS'S HOSPITAL.

JANUARY 21st, 1816.—John Iliff, ætat. twenty-one, admitted with symptoms of incarcerated inguinal hernia; gives the following account of his complaints:—Has had a rupture on the left side from his infancy. He wore a truss for many years, but, this having been broken, he neglected to procure another; and from this time the hernia has generally been down. It was always, however, reducible, and could be returned by moderate pressure: this he used to effect by crossing his legs, and pressing them strongly together. On one occasion this attempt produced considerable pain, and proved ineffectual: it was then reduced by a surgeon.

Yesterday evening, when running violently, he fell; the hernia came down, and he was unable to return it. It increased in size, and became painful. A surgeon, who was sent for, bled him twice, and tried the taxis without effect. Cold was then applied to the tumor, but the patient passed the night in pain, with constant vomiting.

On his admission, the tumor was tense, of a pale-red colour, not very tender to the touch. It distended the scrotum considerably, the testicle being situated at the most inferior and convex part: it was very moveable. The swelling felt partly elastic, like intestine distended with flatus, while other portions communicated the doughy feel of omentum. The neck of the hernia was proportionately thick, and it appeared to lead, with very little obliquity, into the abdomen. Stretching across between this and the hernial tumor, a tense band was distinctly perceptible to the eye. He complained of slight pain in the belly, which was relieved by pressure; his pulse was small and quick, with some hardness; his countenance extremely anxious. No stool since the hernia came down.

The taxis was tried, and persisted in for twenty minutes without avail: he was then put into a warm bath (110), and the attempts at reduction continued. In fifteen minutes, being very faint, he was removed from the bath. Having recovered from this state, a pound of blood was taken from the arm by a large orifice: a tendency to syncope supervened, and the taxis was again had recourse to, but without success. He was now placed in bed; a freezing mixture was applied to the scrotum, and a tobacco glyster administered, (ʒss to ℔ss.) This was soon followed by nausea, and great general debility and distress. The taxis was once more put in practice, and, again failing, general pressure was applied to the tumor, and persevered in for half an hour, with a view of lessening its bulk by expelling the contained flatus. These means proving ineffectual, and the symptoms of incarceration continuing undiminished, the operation was had recourse to at one o'clock.

An incision, commencing a little above the external ring, was continued for about three inches down the tumor, and through the common integuments. The superficial fascia, which was rather thickened, being next divided, exposed that part of the tendon of the exterior abdominal muscle which passes from one pillar of the ring to the other. This had a distinct semilunar figure, and explained the appearance described before the integuments were divided. This formed a partial stricture, and was divided in an oblique direction. An attempt was then made to return the hernia, but another stricture was found to exist higher up. A director was next passed under the superior pillar of the ring, which was cut by a straight incision. After this had been effected, gentle pressure was made upon the tumor, and the hernia rapidly ascended, without the necessity of opening the sac. The patient expressed immediate relief. The wound was closed by a stitch and adhesive plaster: he was replaced in bed.

Ten o'clock, evening.—Vomited immediately after the operation; slept some hours. No stool; no pain in the abdomen, but complains of pain about the chest and scrobiculus cordis; pulse quick; skin hot.

> Enema purgans statim et Ol. Ricini ℥ss. quarta quâque hora donec, &c.

22d, nine o'clock A.M.—Has slept well. Two copious evacuations. Pain at the chest continues, and is attended with cough, which causes the hernia to protrude; abdomen easy.

23d, morning.—Complaint of the chest increased; slight pain in the abdomen; skin hot; bowels confined.

> Mittatur sanguis ad ℥viij.

> Sulphat Magnesiæ, ʒi.; Mist. Ammoniæ acet. ℥ss. quarta quâque hora.

Evening.—No motion: in other respects as before.

> Enema purgans statim.

24th.—Much better; two evacuations. Wound dressed; looking well.

> Pergat.

February 8th.—Discharged cured. (To wear a truss.)

